# Measuring 24-hour Movement Profiles During Pregnancy: Protocol for the 24MOVE Prospective Cohort Study

**DOI:** 10.2196/72828

**Published:** 2025-09-15

**Authors:** Elizabeth Ryan, Alex Asera, Kelley Pettee Gabriel, Rachel Manber, Charles P Quesenberry, Lyndsay A Avalos, Monique M Hedderson, Sylvia E Badon

**Affiliations:** 1 Division of Research Kaiser Permanente Northern California Pleasanton, CA United States; 2 Department of Epidemiology University of Alabama at Birmingham Birmingham, AL United States; 3 Department of Psychiatry and Behavioral Sciences Standford University School of Medicine Stanford, CA United States

**Keywords:** 24-hour movement, physical activity, sleep, pregnancy, sedentary behavior, obesity, gestational diabetes, maternal glucose tolerance, adverse pregnancy outcomes, infant birthweight

## Abstract

**Background:**

Physical activity (PA) during the waking period and sleep during pregnancy may mitigate the increased risk for adverse pregnancy outcomes posed by gestational diabetes mellitus (GDM) and excessive gestational weight gain (GWG) in pregnant individuals with pre-pregnancy overweight and obesity. Recent studies have conceptualized PA, sedentary behavior, and sleep as part of a 24-hour movement framework; however, there is a gap in the knowledge about the relationship between 24-hour movement and pregnancy outcomes.

**Objective:**

This paper describes the study protocol for the 24MOVE study, a prospective cohort study that examines associations between 24-hour movement profiles across pregnancy and maternal glucose tolerance, GWG, and infant birthweight.

**Methods:**

Participants (N=306) were recruited from a large, integrated health care delivery system at 10 weeks’ gestation. In early (10-12 weeks), mid- (20-22 weeks), and late (33-35 weeks) pregnancy, all eligible individuals with a pre-pregnancy BMI of ≥25 kg/m2 completed online surveys collecting information about sociodemographic characteristics and pregnancy symptoms and behaviors, including sleep quality. Participants concurrently wore a research-grade accelerometer for 24 hours per day for 7 consecutive days to capture movement, sedentary behavior, and sleep data. Data from accelerometry will be processed to create 24-hour movement profiles. Pregnancy outcomes will be ascertained from electronic health records (EHRs). We will use compositional data analysis (CoDA) methods, modeling associations of reallocations of time from one component behavior to another at various timepoints with outcomes.

**Results:**

Recruitment began on March 19, 2023, and ended on September 11, 2024. Enrollment was completed on September 19, 2024. Data collection was completed in April 2025. Over an 18-month recruitment period, 2035 individuals were invited to participate, and of those, 306 (15%) eligible participants were enrolled in this study. The enrolled cohort had a median age of 33 (quintile 1 [Q1]-quintile 3 [Q3] 30-36) years and a median BMI of 28.8 (Q1-Q3 26.9-32.7) kg/m². Most participants had private insurance (n=266, 86.9%) and were multiparous (n=232, 75.8%). Analyses are in progress.

**Conclusions:**

The 24MOVE study is designed to address gaps in our knowledge of the impact of 24-hour movement during pregnancy on maternal glucose metabolism, GWG, and other risk factors for childhood obesity, such as a high birthweight. Data from this study will also serve as a rich resource for future investigations of 24-hour movement profiles and behavior substitutions and other perinatal mental and physical health outcomes.

**International Registered Report Identifier (IRRID):**

DERR1-10.2196/72828

## Introduction

A high maternal pre-pregnancy BMI, gestational diabetes mellitus (GDM), and excessive gestational weight gain (GWG; value above the Institute of Medicine recommendations [1]) are prevalent pregnancy conditions that contribute to an in utero environment of overnutrition, leading to significant short- and long-term health ramifications for pregnant people and their children, including increased risks for obesity and diabetes [2-11]. Pregnant individuals with both obesity and excessive GWG have the highest risks of adverse pregnancy outcomes [12], and their children are also at greater risk of childhood obesity [13-15] and a greater BMI [16] in adulthood compared to children exposed to a normal weight and GWG within recommendations in utero.

Physical activity (PA) during the waking period and sleep during pregnancy may mitigate the increased risk for adverse pregnancy and health outcomes posed by GDM and excessive GWG in pregnant individuals with a high pre-pregnancy BMI. When studied as individual behaviors, regular moderate- to vigorous-intensity PA during pregnancy, light-intensity PA, less sedentary behavior, and adequate sleep are associated with pregnancy-specific health benefits [17], including lowering the risk of GDM [18-25], abnormal glucose tolerance [26-29], and excessive GWG [30-36]. Moderate-to vigorous-intensity PA is also associated with a lower risk of large for gestational age (LGA) birthweight [37]. In addition, emerging evidence suggests that sleep regularity, including consistent timing of sleep and wake periods, may be important for optimal health outcomes in adults [38].

Given that PA, sedentary behavior, and sleep are interrelated behaviors in the context of a 24-hour day, recent studies have conceptualized these behaviors as part of a 24-hour movement framework [39,40]. Informed by associations of 24-hour movement profiles and individual component behaviors with physical and mental health outcomes in the general adult population, Canada [41] and Thailand [42] have released “24-Hour Movement Guidelines for Adults,” and Australia is in the process of incorporating a 24-hour approach for their next set of “Physical Activity and Sedentary Guidelines for Adults” [43]. In the United States, the most recent PA guidelines [44] include the recommendation to “sit less, move more, implying that for individuals initiating or increasing PA, replacing sedentary behavior with PA is a beneficial behavior change. Recent research has also highlighted the importance of integrating 24-hour movement guidance with dietary recommendations to support more holistic public health strategies [45]. These recommendations recognize the importance of considering the balance of behaviors in the 24-hour day, as well as behavior change as a replacement of one behavior for another in the context of the 24-hour day, but do not provide specific guidelines during pregnancy, a time when behavior change is common.

Initial studies on 24-hour movement in pregnancy suggest behavior reallocations to maintain or increase moderate- to vigorous-intensity PA may be beneficial for reducing pregnancy-induced increases in cardiometabolic biomarkers [46,47], improving body composition [46,48], and improving cardiorespiratory fitness [48]. In addition, one study found that reallocating time from sleep or sedentary behavior to light-intensity PA in early pregnancy may be beneficial for improving body composition and cardiometabolic biomarkers in late pregnancy [46]. However, these studies have all been exploratory and focused on risk factors for adverse outcomes. Larger, more representative, and longitudinal studies designed to address research questions on 24-hour movement and pregnancy outcomes, rather than risk factors, are needed to guide recommendations in this population.

As previous studies have all been exploratory, the impact of 24-hour movement during pregnancy on maternal glucose metabolism, GWG, and LGA birthweight remains uncertain. To address crucial knowledge gaps, studies with concurrent measurement of sedentary behavior, PA, and sleep throughout pregnancy are essential for informing recommendations and interventions to promote a healthy 24-hour day during pregnancy and optimal pregnancy outcomes.

This paper describes the study protocol for the 24MOVE study, a prospective cohort study that seeks to fill these knowledge gaps by examining associations between 24-hour movement profiles across pregnancy and maternal glucose tolerance, GWG, and infant birthweight. The aims of this study are to (1) examine relationships of 24-hour movement profiles in early and midpregnancy with maternal glucose tolerance in midpregnancy; (2) examine relationships of 24-hour movement profiles in early, mid-, and late pregnancy with GWG, and (3) examine relationships of 24-hour movement profiles in early, mid-, and late pregnancy with infant birthweight.

## Methods

### Study Setting

This study is currently being conducted within Kaiser Permanente Northern California (KPNC), an integrated health care delivery system providing care for more than 66,000 pregnancies (40,000 live births) annually in Northern California. KPNC health plan members are covered by employer-sponsored insurance plans, the California Insurance Exchange, Medicare, and Medicaid. KPNC membership represents the diverse population of the Northern California region in terms of demographic, ethnic, and socioeconomic characteristics, except with respect to income, where members underrepresent the poor and the wealthy [49]. Leveraging the KPNC’s electronic health records (EHRs), participants were identified and recruited from three KPNC medical centers: Oakland, Walnut Creek, and South Sacramento. The Oakland, Walnut Creek, and South Sacramento medical centers represent urban, suburban, and semirural regions across Northern California, providing a geographically and socioeconomically diverse sample.

### Ethical Considerations

All study procedures were reviewed and approved by the KPNC Institutional Review Board (IRB approval number 1998165). Written informed consent was obtained from all participants prior to study enrollment, including consent for use of their data in future analyses. Participants were informed that participation was voluntary and that they could withdraw from the study at any time. All data used in this study were deidentified prior to analysis in order to protect participant privacy and confidentiality. No identifiable personal health information was used in analyses or shared outside the study team. Participants received compensation as follows:

- A US $75 gift card at each of the three study timepoints (early, mid-, and late pregnancy) after completing the study surveys and returning the accelerometer
- A US $25 gift card at each timepoint for completing three time-use and three diet surveys
- A US $20 gift card after completing the feedback survey at the late pregnancy timepoint

### Overview of the Study Design

This prospective cohort study identified newly confirmed pregnancies among KPNC members with pre-pregnancy overweight or obesity through the KPNC’s EHRs. Identified individuals were invited to participate in this study via letter and email invitations. Eligible participants who provided written informed consent completed surveys (copies of study surveys are available in Multimedia Appendix 1) wore an accelerometer on their nondominant wrist for 24 hours per day for 7 days and participated in daily short sleep surveys at three timepoints in pregnancy (early, mid-, and late). During select days of activity and sleep monitoring, participants received links for additional surveys on 24-hour time use and diet. At the late pregnancy timepoint, participants also received an additional survey asking for feedback on their experience in the study, including their motivation for participating, any problems or issues they experienced with any aspect of the study, and suggestions for future research questions that are important to them. Pregnancy outcomes were ascertained from the EHRs.

### Recruitment

Eligible KPNC members at 10 weeks’ gestation (10 weeks 0 days to 10 weeks 6 days) were identified weekly from the EHRs. Identified members received an invitation letter by mail, including a link to an online eligibility survey, administered via Research Electronic Data Capture (REDCap), to confirm eligibility for the study. After completing the eligibility survey, eligible individuals who chose to participate in the study proceeded to complete informed consent. In cases of nonresponse to the mailed letter invitation after a week, an email study invitation was sent from the study email address. If there was still no response to the email after a week, a research assistant followed up once by phone. Members who did not respond to any of these invitations were not contacted further. Those who initiated but did not complete the baseline survey received two reminders via email through REDCap (1 reminder per week) after initiating the survey. If the survey was not completed after the reminders were sent, the individual was not contacted further.

### Eligibility Criteria

As summarized in Table 1, potential participants were identified using initial EHR criteria, and eligibility was confirmed with an online eligibility survey.

**Table 1:** Eligibility criteria for the 24MOVE study.

Eligibility criterion	EHRs^a^	Eligibility survey
KPNC^b^ membership	X^c^	X
Currently pregnant	X	X
Receiving care at Kaiser Permanente Oakland, South Sacramento, or the Walnut Creek Medical Center	X	—^d^
Singleton pregnancy	X	X
≤12 weeks’ gestation (10 weeks 0 days to 12 weeks 6 days)	X	X
Age ≥18 years	X	X
Pre-pregnancy BMI ≥25 kg/m² (based on closest weight measured within 12 months prior to pregnancy)	X	—
No diabetes diagnosis in the 12 months before pregnancy	X^e^	X
Not currently on bed rest or activity restriction (to exclude those with extreme limitations on their PA^f^)	—	X
No diagnosis or treatment of a sleep disorder in the 12 months before pregnancy (to exclude those with disrupted sleep patterns)	X^g^	X
No clinically significant insomnia symptoms (Insomnia Severity Index [50,51] <15, to exclude those with disrupted sleep patterns)	—	X

^a^EHR: electronic health record.

^b^KPNC: Kaiser Permanente Northern California.

^c^Applicable.

^d^Not applicable.

^e^Identified using the KPNC Diabetes Registry [52]

^f^PA: physical activity.

^g^Identified using International Classification of Diseases (ICD) codes F51 and G47.

### Overview of Data Collection

At each of the three data collection timepoints—early pregnancy (10 weeks 0 days to 12 weeks 6 days), midpregnancy (20 weeks 0 days to 22 weeks 6 days), and late pregnancy (33 weeks 0 days to 35 weeks 6 days)—participants completed a set of surveys administered through REDCap (copies of study surveys are available in Multimedia Appendix 1), wore a Centrepoint Insight accelerometer (ActiGraph) on their nondominant wrist for 24 hours per day for 7 days, and participated in short daily sleep surveys. The surveys at each timepoint covered a broad range of topics, including PA levels, pregnancy-related symptoms, substance use, perceived social support, depressive and anxiety symptoms, perceived stress levels, quality of life, and sociodemographic characteristics (Table 2). At the early pregnancy timepoint, participants additionally reported whether it was their first pregnancy, the number of children currently residing with them, the birthdate of each child, their typical waking times on weekdays and weekends since the beginning of pregnancy, their gender identity, their racial and ethnic identities, educational attainment, the total annual household income, the number of individuals supported by this income, marital status, and wrist size. Participants’ address and contact information were confirmed, by phone or as part of the online survey, at each study timepoint. After survey completion at each timepoint, accelerometers were mailed to participants, along with a prepaid return envelope. Participants were provided detailed written instructions on how to properly wear, care for, and return the accelerometer, including a frequently asked question (FAQ) section covering common concerns. Participants received a link to a Consensus Sleep Diary via a text message at their usual wake time (as indicated on the early pregnancy survey) for 7 consecutive days during the accelerometer wear period. If typical wake times were not provided by a participant, surveys were sent at 8:00 AM by default. On 3 random days during the 7-day period of accelerometer wear, participants received links via email for additional surveys for 24-hour recall of time use and diet. After delivery, maternal glucose tolerance–screening results, GWG, and infant birthweight will be abstracted from prenatal care and delivery records in the EHRs.

**Table 2:** Overview of data collection at each study timepoint.

Measure			Instrument		Study data collection timepoint				
					Early pregnancy (10-12 weeks’ gestation)		Midpregnancy (20-22 weeks’ gestation)		Late pregnancy (33-35 weeks’ gestation)
**24-hour movements**									
	24-hour accelerometry	Centrepoint Insight watch		X^a^ (7 consecutive days)		X (7 consecutive days)		X (7 consecutive days)	
	In-bed duration, sleep duration, sleep quality, napping	Consensus Sleep Diary [53]		X (7 consecutive days)		X (7 consecutive days)		X (7 consecutive days)	
	PA^b^ and sedentary behavior	Modified PPAQ^c^ [54]		X		X		X	
	Time use	ACT24^d^ [55]		X (3 random days)		X (3 random days)		X (3 random days)	
	PA before pregnancy	Stanford Leisure-Time Activity Categorical Item (L-Cat) [56,57]		X		—^e^		—	
	Usual weekday/weekend wakeup times	—		X		—		—	
**Pregnancy symptoms and behaviors**									
	Nausea and vomiting	Modified PUQE^f^ index [58]		X		X		X	
	Tobacco, alcohol, and marijuana use	—		X		X		X	
	Diet	ASA24^g^ [59]		X (3 random days)		X (3 random days)		X (3 random days)	
**Mental health and social support during pregnancy**									
	Perceived social support	Multidimensional Scale of Perceived Social Support [60,61]		X		X		X	
	Perceived stress	PSS-10^h^ [62]		X		X		X	
	Depressive symptoms	PHQ-8^i^ [63]		X		X		X	
	Anxiety symptoms	GAD-7^j^ [64]		X		X		X	
	Quality of life	QOL-GRAV^k^ [65]		X		X		X	
**Sociodemographic characteristics**									
	Gravidity, number of children living with the participant, birthdates of those children	—		X		—		—	
	Gender identity	—		X		—		—	
	Racial and ethnic identities	—		X		—		—	
	Education	—		X		—		—	
	Employment status	—		X		X		X	
	Household income, number of people supported by this income	—		X		—		—	
	Marital status	—		X		—		—	

^a^Applicable.

^b^PA: physical activity.

^c^PPAQ: Pregnancy Physical Activity Questionnaire.

^d^ACT24: Activities Collected over Time over 24 hours.

^e^Not applicable.

^f^PUQE: Pregnancy-Unique Quantification of Emesis and Nausea.

^g^ASA24: Automated Self-Administered 24-Hour Dietary Assessment.

^h^PSS-10: 10-item Perceived Stress Scale.

^i^PHQ-8: 8-item Patient Health Questionnaire.

^j^GAD-7: 7-item General Anxiety Disorder Scale.

^k^QOL-GRAV: Quality of Life Gravidarum questionnaire.

### Data Processing and Creation of 24-hour Movement Profiles Using Accelerometer Data

#### Accelerometer Wear Time

Raw accelerometer data will be sampled at 32 Hz, downloaded using the Centrepoint software platform (ActiGraph), and processed with R (R Foundation for Statistical Computing) [66]. Vector magnitude counts, summarized for each minute (60-second epoch) of data collection, will be extracted. Nonwear time will be identified using the Choi algorithm [67-69], where nonwear is defined as an interval of ≥90 consecutive minutes with 0 vector magnitude counts/minute, allowing up to 2 minutes of nonzero counts during the 30-minute window before or after that interval. Any nonzero vector magnitude counts (except within the allowed short intervals) will be considered wear time. Valid accelerometer data will be defined as ≥10 hours of waking wear time in a 24-hour period for ≥4 days.

#### Sleep Opportunity Period

The Tracy bed rest algorithm will be used to estimate sleep opportunity periods [70]. Uniaxial counts, summarized for each minute (60-second epoch) of data collection, will be extracted. Wear time will be divided into 60-minute time blocks; counts per minute will be averaged for each block. The algorithm, using a 4-step decision tree process, identifies transition times for getting in and out of bed, with thresholds of 400 counts per minute for getting in bed and 1500 counts per minute for getting out of bed. Minutes between identified in-bed and out-of-bed times will be classified as the sleep opportunity period and the remaining minutes as wake time. The duration of the sleep opportunity period for each 24-hour period will be calculated and averaged across valid days, with daily averages for sleep opportunity period duration considered as exposures in the 24-hour movement framework. Daytime naps will be separately reported via the Consensus Sleep Diary and will be considered as an additional component of 24-hour movement profiles in secondary analyses. We will also use self-reported measures of sleep quality from sleep diaries as potential effect modifiers in analyses.

#### Light-Intensity PA, Moderate- to Vigorous-Intensity PA, and Sedentary Behavior

We will use the 2-regression algorithms developed by Hibbing et al [71] to categorize the waking period by intensity. Raw acceleration data for each second of identified waking wear time (1-second epochs) will be extracted. Each 1-second epoch will be classified as sedentary or at least light-intensity PA. Sedentary 1-second epochs will be assigned a metabolic equivalent of task (MET) value of 1.25 METs. Epochs classified as light- or higher-intensity PA will be further categorized as continuous walk/run or intermittent activity, regression equations will predict a MET value for each 1-second epoch, and then MET values will be averaged over each minute. Minutes of waking wear time will then be classified as sedentary (≤1.5 METs), light intensity (1.6-2.9 METs), or moderate to vigorous intensity (≥3 METs). Daily averages for light-intensity PA duration, moderate- to vigorous-intensity PA duration, and sedentary behavior duration will be calculated by summing the times per day and averaging across valid days.

#### 24-hour Movement Profiles

Participants’ 24-hour movement profiles will be created by normalizing the sleep opportunity period, sedentary behavior, light-intensity PA, and moderate- to vigorous-intensity PA durations to a 24-hour period.

### Survey Data: 24-hour Movement

#### Self-Reported PA and Sedentary Behavior

The self-reported PA and sedentary behavior levels of participants will be assessed using the Pregnancy Physical Activity Questionnaire (PPAQ) [54]. The PPAQ was developed based on commonly reported physical activities in pregnant women and is a validated tool for assessing PA levels during pregnancy [54]. We used a modified version of the PPAQ that expands on leisure time behaviors to include additional activities (eg, weightlifting, yoga/Pilates/stretching, and team sports). We will use the PPAQ to provide contextual information about activity domains to complement accelerometer data. In scoring, activities reported will be matched to their assigned MET values, which will then be summed across categories to calculate total MET-hours per week or day by intensity of behavior and by domain.

#### 24-hour Time Use

The Activities Collected over Time over 24 hours (ACT24) [55] instrument will be used to collect detailed data on each participant’s activities over the previous 24-hour day, including PA, sedentary behavior, and sleep. The ACT24 is a valid tool for accurately capturing time spent in the previous day’s activities across multiple intensities and domains [55,72,73]. Data from the ACT24 will complement accelerometer data by providing contextual information on the types of activities and domains not captured by movement sensors alone.

### Survey Data: Pregnancy Symptoms and Additional Behaviors

#### Pregnancy Nausea and Vomiting Symptoms

The modified Pregnancy-Unique Quantification of Emesis and Nausea (PUQE) index is a validated tool for measuring the severity of nausea and vomiting during pregnancy [58,74]. It assesses the frequency and intensity of nausea and vomiting and their impact on daily life. Responses are summed to obtain a total score ranging from 3 to 15, with a higher score indicating more severe nausea and vomiting symptoms during pregnancy.

#### Diet

The Automated Self-Administered 24-Hour Dietary Assessment (ASA24) is a web-based recall tool for collecting detailed dietary intake data over a 24-hour period that has been validated for estimating dietary intake in multiple populations [59,75,76]. The ASA24 generates comprehensive reports and analysis files on nutrient intake and food group consumption based on user inputs.

### Survey Data: Mental Health and Social Support During Pregnancy

#### Perceived Social Support

The Multidimensional Scale of Perceived Social Support [60,61] measures perceived support from family, friends, and significant others. It is a widely validated and reliable tool across various populations [60,61,77]. The scale consists of 12 questions scored on a 7-point Likert scale ranging from 1 (very strongly disagree) to 7 (very strongly agree). Scores are calculated for overall social support and within each subscale (family, friends, significant other). Calculated scores range from 1 to 7, with higher scores indicating greater perceived social support.

#### Perceived Stress

The 10-item Perceived Stress Scale (PSS-10) [62] is the most widely used psychological instrument for measuring the perception of stress and has been validated in diverse populations and in perinatal women [78]. Scores range from 0 to 40, with 14 or greater signifying moderate-to-severe perceived stress.

#### Depressive Symptoms

Depressive symptoms will be measured using the 8-item Patient Health Questionnaire (PHQ-8) [63]. The PHQ-8 has been validated in many studies as an instrument for screening for depression, with high sensitivity (>88%) and specificity (>88%) in obstetric patients [63,79]. The PHQ-8 is also a valid tool to establish depression severity and outcome [80]. The 8-question screener scores range from 0 to 24. A score of 1-4 suggests minimal depression; 5-9, mild depression; 10-14, moderate depression; 15-19, moderately severe depression; and 20-24, severe depression.

#### Anxiety Symptoms

The 7-item General Anxiety Disorder Scale (GAD-7) [64] has been validated in prenatal populations and will be used to measure anxiety symptoms [64,81]. Scores range from 0 to 21, and scores of 10-21 are categorized as clinically significant anxiety symptoms.

#### Pregnancy-Related Quality of Life

The Quality of Life Gravidarum questionnaire (QOL-GRAV) is a validated tool for assessing the well-being of pregnant women across physical, emotional, and social aspects [1,65]. Overall scores range from 15 to 35, with a lower score reflecting a higher quality of life during pregnancy.

### Pregnancy Outcomes

Pregnancy outcomes, including glucose tolerance, GWG, and infant birthweight, will be identified from EHR data. The KPNC maintains a virtual data warehouse where data from EHRs are aggregated on a daily basis with rigorous quality control and standardization of data for consistency and accuracy across multiple data sources. Outcomes will be extracted from this database.

#### Maternal Glucose Tolerance

GDM screening at the KPNC consists of a 50 g glucose challenge test for all pregnant patients. If the glucose challenge test results are abnormal (≥140 mg/dL), a subsequent 100 g, 3-hour oral glucose tolerance test is administered. GDM will be diagnosed according to Carpenter-Coustan criteria [82]. Glucose tolerance–screening results and GDM diagnoses will be abstracted from the EHRs. Glucose challenge test results (continuous glucose levels and dichotomized as normal [<140 mg/dL] vs abnormal [≥140 mg/dL]) will be examined as outcomes in the main analyses. In exploratory analyses, we will examine the GDM diagnosis as an outcome.

#### Gestational Weight Gain

Weight measurements will be taken and recorded during each prenatal visit in the EHRs. The total GWG will be calculated as the difference between the final pregnancy weight predelivery (within 3 weeks of delivery) and the pre-pregnancy weight (the most recent clinically measured pre-pregnancy weight within 6 months before the last menstrual period or for women lacking a measured pre-pregnancy weight, the earliest clinically measured pregnancy weight before 10 weeks’ gestation). We will use standardized GWG-for-gestational age charts to calculate GWG z-scores to account for the correlation between the GWG and the gestational age at delivery [83,84]. GWG z-scores will be used as continuous outcomes in the main analyses. The average weekly trimester-specific GWG for the second and third trimesters will also be calculated. The trimester-specific GWG will be categorized using Institute of Medicine recommendations (below, within, or above recommendations) [85]. We will examine the trimester-specific GWG as a continuous outcome, stratified by pre-pregnancy BMI category, and as a categorical outcome.

#### Infant Birthweight

The birthweight is measured at the time of delivery and recorded in the EHR. We will use gestational age data from the EHR in conjunction with national sex-specific birthweight curves to identify birthweight-for-gestational age z-scores and the LGA birthweight [86]. We will examine birthweight as a continuous outcome in the main analyses. In exploratory analyses, we will examine the LGA birthweight as an outcome.

### Sample Size

Given a sample size of 300, there is sufficient power (0.80) to detect an additional 2.1%-2.4% in the proportion of variance in glucose-screening results, GWG, and birthweight explained by the duration of each behavior (sleep, sedentary behavior, light-intensity PA, or moderate- to vigorous-intensity PA) across the expected ranges in R^2^ for a set of confounders (5%-20%; 15 confounders assumed, α=.05, *F*-test). In exploratory analyses, the minimum detectable relative risk of GDM, excessive GWG, and LGA per 1 SD increase in a component behavior is 1.70, 1.12, and 1.60, respectively (power=0.80, α=.05, 2-sided test).

### Planned Statistical Analyses

We will use a compositional data analysis (CoDA) approach for all analyses. We will apply isometric log-ratio transformations to normalized 24-hour movement profiles (the proportion of the 24-hour day spent in the sleep opportunity period, sedentary behavior, light-intensity PA, and moderate- to vigorous-intensity PA) to obtain three transformed variables that capture the codependence and collinearity between these behaviors [87-89]. To examine how hypothetical behavior substitutions within the 24-hour day during pregnancy impact outcomes, we will use a compositional isotemporal substitution approach [90] to model the predicted change in pregnancy outcomes when reallocating up to 60 minutes of time from one component behavior to another at various timepoints. We will assess associations of hypothetical behavior substitutions in early and midpregnancy with glucose tolerance outcomes (aim 1); in early, mid-, and late pregnancy with GWG outcomes (aim 2); and in early, mid-, and late pregnancy with birthweight outcomes (aim 3).

We will use linear regression models to model relationships with continuous outcomes (glucose challenge test results, GWG, birthweight) and log-binomial regression models to model relationships with dichotomous outcomes (abnormal glucose challenge test results, GDM diagnosis, GWG above recommendations, LGA birthweight). All models will be adjusted for a priori–identified confounding variables (eg, maternal age, race and ethnicity, education, income, employment, pre-pregnancy BMI, parity, diet, depression and anxiety symptoms, stress, smoking and alcohol use, and severity of nausea and vomiting). We will assess potential multicollinearity using variance inflation factors and exclude covariates from the models, as necessary. Departures from linearity will be addressed by the addition of higher order (eg, quadratic) terms. Departures from model assumptions will be assessed via diagnostic plots of weighted residuals. Missing data will be assessed, and if necessary, missing data in covariates and outcomes will be handled using standard multiple imputation methods for longitudinal data analysis, namely multivariate imputation by chained equations (MICE), which has also been referred to as fully conditional specification (FCS).

## Results

### Study Timeline

Recruitment began on March 19, 2023, and ended on September 11, 2024. Enrollment was completed on September 19, 2024. Data collection was completed in April 2025.

### Recruited Cohort

**Figure 1:**
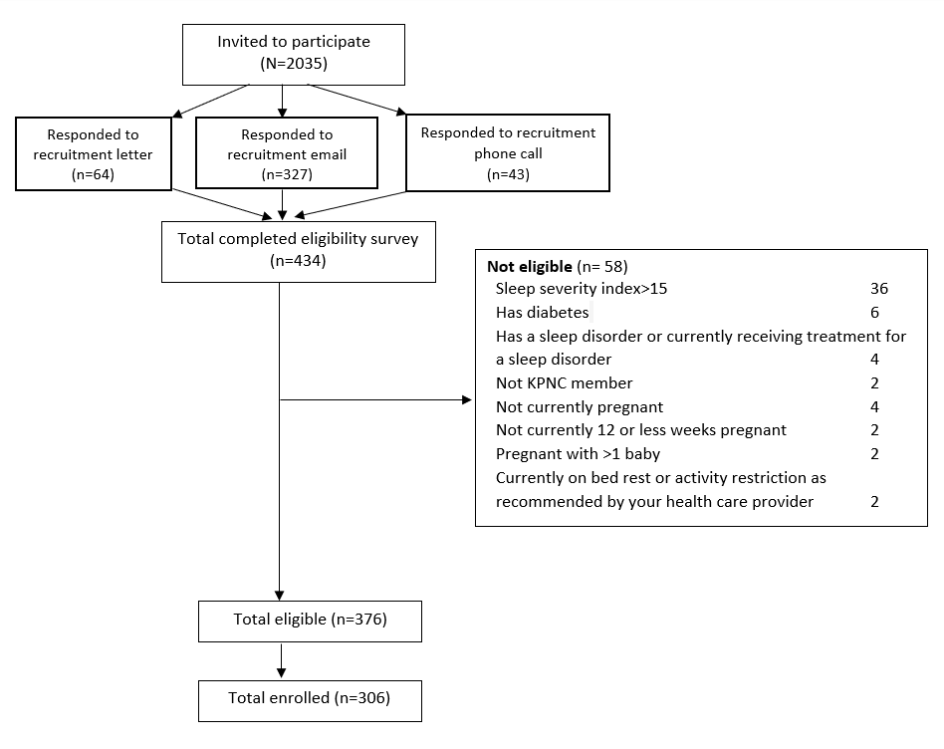
Summary of recruitment for the 24MOVE study. In total, 2035 participants were invited to participate, 434 (21.3%) responded and completed an eligibility survey, 376 (86.6%) were eligible for the study, and 306 (81.4%) were enrolled. KPNC: Kaiser Permanente Northern California.

Over an 18-month recruitment period, we invited 2035 KPNC members to participate in this study (Figure 1). Responses to the invitation were received via recruitment emails (n=327, 16.1%), recruitment letters (n=64, 3.1%), and recruitment phone calls (n=43, 2.1%). Of those invited, 434 (21.3%) individuals completed the eligibility survey, and 376 (86.6%) met the eligibility criteria. The most prevalent reason for ineligibility was clinically significant insomnia symptoms (n=36, 8.3%). A total of 306 (81.4%) participants consented to participate in the study and were enrolled.

Enrolled participants had a median age of 33 (quintile 1 [Q1]-quintile 3 [Q3] 30-36) years with a median pre-pregnancy BMI of 28.8 (Q1-Q3 26.9-32.7) kg/m^2^ (Tables 3 and 4). Among the 306 participants, 129 (42.2%) were non-Hispanic White, 61 (19.9%) were Hispanic, 48 (15.7%) were Asian, and 24 (7.8%) were non-Hispanic Black. Most participants had private insurance (n=266; 86.9%) and were multiparous (n=232; 75.8%). Pre-pregnancy anxiety and depression had a prevalence of 19%-22% (n=58-68), while hypertension and diabetes were uncommon (reported by 0-12, 0%-4%, participants). Comparing sociodemographic and pre-pregnancy health characteristics between invited participants and the subset that were eligible and enrolled in this study, the enrolled cohort underrepresented non-Hispanic Black, Asian, and Hispanic participants and those with Medicaid insurance compared to the invited group. Other characteristics (age, parity, and pre-pregnancy health) were similar between the invited and enrolled participants.

**Table 3:** Comparison of sociodemographic characteristics between subsets of participants invited to the 24MOVE study.

Characteristics			Invited (N=2035)		Completed eligibility survey (n=434)		Eligible (n=376)		Enrolled (n=306)
Age (years), median (Q1^a^-Q3^b^)			32 (28-35)		33 (30-36)		33 (30-36)		33 (30-36)
**Race and ethnicity, n (%)**									
	Asian	424 (20.8)		70 (16.1)		61 (16.2)		50 (16.3)	
	Non-Hispanic Black	283 (13.9)		41 (9.4)		33 (8.8)		25 (8.2)	
	Hispanic	497 (24.4)		100 (23.0)		82 (21.8)		61 (19.9)	
	Multiracial	170 (8.4)		42 (9.7)		35 (9.3)		30 (9.8)	
	Non-Hispanic White	560 (27.5)		158 (36.4)		147 (39.1)		128 (41.8)	
	Other	63 (3.1)		12 (2.7)		9 (2.3)		4 (1.3)	
	Missing	38 (1.9)		11 (2.5)		9 (2.3)		8 (2.6)	
**Medicaid insurance, n (%)**									
	Insured	333 (16.4)		44 (10.1)		36 (9.6)		28 (9.2)	
	Missing	90 (4.4)		18 (4.1)		13 (3.5)		12 (3.9)	
**Nulliparous, n (%)**									
	Yes	316 (15.5)		67 (15.4)		58 (15.4)		45 (14.7)	
	Missing	145 (7.1)		34 (7.8)		32 (8.5)		27 (8.8)	

^a^Q1: quintile 1.

^b^Q3: quintile 3.

**Table 4:** Comparison of pre-pregnancy health characteristics between subsets of participants invited to the 24MOVE study.

Characteristics	Invited (N=2035)	Completed eligibility survey (n=434)	Eligible (n=376)	Enrolled (n=306)
BMI (kg/m^2^), median (Q1^a^-Q3^b^)	30.4 (27.4-34.9)	29.2 (27.0-33.5)	29.1 (26.9-33.1)	28.8 (26.9-32.7)
Anxiety diagnosis, n (%)	396 (19.5)	101 (23.3)	89 (23.7)	68 (22.2)
Depression diagnosis, n (%)	339 (16.7)	88 (20.3)	72 (19.1)	57 (18.6)
Hypertension diagnosis, n (%)	95 (4.7)	19 (4.4)	15 (4.0)	12 (3.9)
Diabetes diagnosis, n (%)	11 (0.5)	2 (0.5)	1 (0.3)	1 (0.3)

^a^Q1: quintile 1.

^b^Q3: quintile 3.

## Discussion

### Summary

The 24MOVE study is designed to address gaps in our knowledge regarding the impact of 24-hour movement during pregnancy on maternal glucose metabolism, GWG, and other risk factors for childhood obesity, such as a high birthweight. Data from this study will serve as a rich resource for future investigations of 24-hour movement profiles and behavior substitutions and perinatal mental and physical health outcomes, in addition to the aims of this study. As a growing number of countries release “24-Hour Movement Guidelines for Adults,” the results from this study will contribute to the body of literature that will guide future 24-hour movement guidelines for pregnant individuals.

### Strengths and Limitations

Strengths of the 24MOVE study include prospective data collection using wearable devices and self-reported measures for all component behaviors during the 24-hour day at multiple timepoints across pregnancy, allowing for the assessment of the duration, intensity, and domain of PA and sedentary behavior and the duration and quality of sleep, including napping. In addition, all data collection is remote, and outcomes are ascertained from EHRs, decreasing the participant burden. Our planned statistical analysis approach using CoDA addresses limitations of conventional statistical approaches, including all mutually exclusive component behaviors of the 24-hour day [91-94]. We will also assess a variety of important confounders and covariates in our data collection and include them in analysis models, as well as assess and address missing data using statistical approaches to maximize available data.

Several potential limitations should also be considered. Although more than 50% of our enrolled study cohort are participants from minoritized racial and ethnic groups, these groups are underrepresented compared to those invited to participate in the study. In addition, those with Medicaid insurance (a proxy for low income) are also underrepresented in the enrolled cohort. Our cohort may not be representative of pregnancies with pre-pregnancy overweight or obesity across the United States. We did not collect data on whether the pregnancy was planned, which may influence pregnancy-related behaviors and outcomes. Although we attempted to minimize the participant burden as much as possible by streamlining surveys and accelerometer protocols, there is a risk of participant fatigue due to multiple data collection modalities over the three timepoints of data collection. During follow-up data collection, we monitored participant engagement and loss to follow-up, amending study procedures to address identified issues if necessary. We will also carefully consider representativeness and loss to follow-up in our analyses and apply appropriate statistical methods to mitigate some of these concerns.

### Dissemination of Results

Results from the 24MOVE study will be disseminated widely via publication in peer-reviewed journals and presentations at national and international conferences.

### Conclusion

The 24MOVE study seeks to enhance our understanding of how 24-hour movement throughout pregnancy influences maternal glucose regulation, the GWG, and key risk factors for childhood obesity, including a high birthweight. Furthermore, the study will offer a comprehensive data resource for future research on movement patterns, behavioral changes, and associations with perinatal mental and physical health outcomes.

## Appendix

Multimedia Appendix 1 First trimester survey and feedback survey.

## Abbreviations

ACT24: Activities Collected over Time over 24 hours
ASA24: Automated Self-Administered 24-hour Dietary Assessment
CoDA: compositional data analysis
EHR: electronic health record
GAD-7: 7-item Generalized Anxiety Disorder Scale
GDM: gestational diabetes mellitus
GWG: gestational weight gain
KPNC: Kaiser Permanente Northern California
LGA: large for gestational age
MET: metabolic equivalent of task
PA: physical activity
PHQ-8: 8-item Patient Health Questionnaire
PPAQ: Pregnancy Physical Activity Questionnaire
PSS-10: 10-item Perceived Stress Scale
PUQE: Pregnancy-Unique Quantification of Emesis and Nausea
Q1: quintile 1
Q3: quintile 3
QOL-GRAV: Quality of Life Gravidarum questionnaire
REDCap: Research Electronic Data Capture

## Data Availability

Deidentified individual participant data and supporting documentation will be made publicly available in a data repository upon publication, in accordance with funder requirements. The code used for statistical analyses will be provided as a supplement to the published manuscript or made available in the same repository.
