# Supplementary material for: Measuring 24-hour Movement Profiles During Pregnancy: Protocol for the 24MOVE Prospective Cohort Study
Source: JMIR Res Protoc. 2025 Sep 15;14:e72828. doi: 10.2196/72828 (PMC12481144; doi:10.2196/72828)
Supplement: Multimedia Appendix 1 [file resprot_v14i1e72828_app1.pdf]

Supplemental Material

Protocol for the 24MOVE Study: a Prospective Cohort Study Measuring 24-Hour Movement Profiles During Pregnancy

Table of Contents

**I.**	**First Trimester Survey**	Pages 2-20
	**Section A: Children**	Page 2
	**Section B: Physical Activity**	Pages 3-9
	**Section C: Pregnancy Symptoms**	Page 10
	**Section D: Alcohol and Smoking**	Pages 11-12
	**Section E: Social Support**	Page 13
	**Section F: Perceived Stress**	Page 14
	**Section G: Depression**	Page 15
	**Section H: Anxiety**	Page 16
	**Section I: Quality of Life**	Page 17
	**Section J: Demographics**	Pages 18-19
	**Section K: Confirmation of Contact**	Page 20
**I.**	**Feedback Survey**	Pages 21-22

First Trimester Survey

24-Hour Movement and Pregnancy Health (24MOVE) Study

Thank you for your participation in the 24-Hour Movement and Pregnancy Health Study. This survey includes questions about your medical history, physical activity, sleep, and current health. It also includes some background questions about you and your children. It is very important that you tell us about yourself honestly. There are no right or wrong answers. If you have any questions, you may contact us at 24MOVEStudy@kp.org or (833) 796-2557. Thank you.

Section A: Your Children

Is this your first pregnancy?

◯ Yes
◯ No

How many children currently live with you? __________________________________

What are the birthdates for each of the children living with you? __________________________________

____

____

____

____

____

____

____

____

____

Section B: This section asks about your physical activity before and during your current pregnancy.

Think about your physical activity before you became pregnant.

During the year before you became pregnant, which statement best describes the kinds of physical activity you usually did? Do not include the time you spent working at a job. Please read all six statements before selecting one.

◯ I did not do much physical activity. I mostly did things like watching television, reading, playing cards, or playing computer games. Only occasionally, no more than once or twice a month, did I do anything more active such as going for a walk or playing tennis.
◯ Once or twice a week, I did light activities such as getting outdoors on the weekends for an easy walk or stroll. Or once or twice a week, I did chores around the house such as sweeping floors or vacuuming.
◯ About three times a week, I did moderate activities such as brisk walking, swimming, or riding a bike for about 15-20 minutes each time. Or about once a week, I did moderately difficult chores such as raking or mowing the lawn for about 45-60 minutes. Or about once a week, I played sports such as softball, basketball, or soccer for about 45-60 minutes.
◯ Almost daily, that is five or more times a week, I did moderate activities such as brisk walking, swimming, or riding a bike for 30 minutes or more each time. Or about once a week, I did moderately difficult chores or played sports for 2 hours or more.
◯ About three times a week, I did vigorous activities such as running or riding hard on a bike for 30 minutes or more each time.
◯ Almost daily, that is, five or more times a week, I did vigorous activities such as running or riding hard on a bike for 30 minutes or more each time.

Since the beginning of your pregnancy, when you were NOT at work, how much time did you usually spend:

Preparing meals (cook, set table, wash dishes)

◯ None
◯ Less than 1/2 hour per day
◯ 1/2 to almost 1 hour per day
◯ 1 to almost 2 hours per day
◯ 2 to almost 3 hours per day
◯ 3 or more hours per day

Dressing, bathing, feeding children while you are sitting

◯ None
◯ Less than 1/2 hour per day
◯ 1/2 to almost 1 hour per day
◯ 1 to almost 2 hours per day
◯ 2 to almost 3 hours per day
◯ 3 or more hours per day

Dressing, bathing, feeding children while you are standing

◯ None
◯ Less than 1/2 hour per day
◯ 1/2 to almost 1 hour per day
◯ 1 to almost 2 hours per day
◯ 2 to almost 3 hours per day
◯ 3 or more hours per day

Playing with children while you are sitting or standing

◯ None
◯ Less than 1/2 hour per day
◯ 1/2 to almost 1 hour per day
◯ 1 to almost 2 hours per day
◯ 2 to almost 3 hours per day
◯ 3 or more hours per day

Playing with children while you are walking or running

◯ None
◯ Less than 1/2 hour per day
◯ 1/2 to almost 1 hour per day
◯ 1 to almost 2 hours per day
◯ 2 to almost 3 hours per day
◯ 3 or more hours per day

Carrying children

◯ None
◯ Less than 1/2 hour per day
◯ 1/2 to almost 1 hour per day
◯ 1 to almost 2 hours per day
◯ 2 to almost 3 hours per day
◯ 3 or more hours per day

Taking care of an older adult

◯ None
◯ Less than 1/2 hour per day
◯ 1/2 to almost 1 hour per day
◯ 1 to almost 2 hours per day
◯ 2 to almost 3 hours per day
◯ 3 or more hours per day

Sitting and reading, writing, typing or doing other desk work, while not at work

◯ None
◯ Less than 1/2 hour per day
◯ 1/2 to almost 1 hour per day
◯ 1 to almost 2 hours per day
◯ 2 to almost 3 hours per day
◯ 3 or more hours per day

Since the beginning of your pregnancy, when you were NOT at work, how much time did you usually spend:

Sitting and watching TV, a movie or video clip, while not at work

◯ None
◯ Less than 1/2 hour per day
◯ 1/2 to almost 2 hours per day
◯ 2 to almost 4 hours per day
◯ 4 to almost 6 hours per day
◯ 6 or more hours per day

Sitting and talking (either in person or on the phone or other device) or texting, while not at work

- ◯ None
- ◯ Less than 1/2 hour per day
- ◯ 1/2 to almost 2 hours per day
- ◯ 2 to almost 4 hours per day
- ◯ 4 to almost 6 hours per day
- ◯ 6 or more hours per day

Playing with pets

- ◯ None
- ◯ Less than 1/2 hour per day
- ◯ 1/2 to almost 1 hour per day
- ◯ 1 to almost 2 hours per day
- ◯ 2 to almost 3 hours per day
- ◯ 3 or more hours per day

Light cleaning (make beds, laundry, iron, put things away)

- ◯ None
- ◯ Less than 1/2 hour per day
- ◯ 1/2 to almost 1 hour per day
- ◯ 1 to almost 2 hours per day
- ◯ 2 to almost 3 hours per day
- ◯ 3 or more hours per day

Shopping (for food, clothes, or other items)

- ◯ None
- ◯ Less than 1/2 hour per day
- ◯ 1/2 to almost 1 hour per day
- ◯ 1 to almost 2 hours per day
- ◯ 2 to almost 3 hours per day
- ◯ 3 or more hours per day

Heavier cleaning (vacuum, mop, sweep, wash windows)

- ◯ None
- ◯ Less than 1/2 hour per week
- ◯ 1/2 to almost 1 hour per week
- ◯ 1 to almost 2 hours per week
- ◯ 2 to almost 3 hours per week
- ◯ 3 or more hours per week

Mowing lawn while on a riding mower

- ◯ None
- ◯ Less than 1/2 hour per week
- ◯ 1/2 to almost 1 hour per week
- ◯ 1 to almost 2 hours per week
- ◯ 2 to almost 3 hours per week
- ◯ 3 or more hours per week

Mowing lawn using a walking mower, raking, gardening

◯ None
◯ Less than 1/2 hour per week
◯ 1/2 to almost 1 hour per week
◯ 1 to almost 2 hours per week
◯ 2 to almost 3 hours per week
◯ 3 or more hours per week

Going Places ...
Since the beginning of your pregnancy, how much time did you usually spend:

Walking slowly to go places (such as to the bus, work, visiting) not for fun or exercise

◯ None
◯ Less than 1/2 hour per day
◯ 1/2 to almost 1 hour per day
◯ 1 to almost 2 hours per day
◯ 2 to almost 3 hours per day
◯ 3 or more hours per day

Walking quickly to go places (such as to the bus, work, or school) not for fun or exercise

◯ None
◯ Less than 1/2 hour per day
◯ 1/2 to almost 1 hour per day
◯ 1 to almost 2 hours per day
◯ 2 to almost 3 hours per day
◯ 3 or more hours per day

Driving or riding in a car or on public transportation

◯ None
◯ Less than 1/2 hour per day
◯ 1/2 to almost 1 hour per day
◯ 1 to almost 2 hours per day
◯ 2 to almost 3 hours per day
◯ 3 or more hours per day

For Fun or Exercise
Since the beginning of your pregnancy, how much time did you usually spend:

Walking slowly for fun or exercise

◯ None
◯ Less than 1/2 hour per week
◯ 1/2 to almost 1 hour per week
◯ 1 to almost 2 hours per week
◯ 2 to almost 3 hours per week
◯ 3 or more hours per week

Walking more quickly for fun or exercise

◯ None
◯ Less than 1/2 hour per week
◯ 1/2 to almost 1 hour per week
◯ 1 to almost 2 hours per week
◯ 2 to almost 3 hours per week
◯ 3 or more hours per week

Walking quickly up hills for fun or exercise

◯ None
◯ Less than 1/2 hour per week
◯ 1/2 to almost 1 hour per week
◯ 1 to almost 2 hours per week
◯ 2 to almost 3 hours per week
◯ 3 or more hours per week

Jogging

◯ None
◯ Less than 1/2 hour per week
◯ 1/2 to almost 1 hour per week
◯ 1 to almost 2 hours per week
◯ 2 to almost 3 hours per week
◯ 3 or more hours per week

Prenatal exercise class

◯ None
◯ Less than 1/2 hour per week
◯ 1/2 to almost 1 hour per week
◯ 1 to almost 2 hours per week
◯ 2 to almost 3 hours per week
◯ 3 or more hours per week

Swimming, water walking, or water exercise (DO NOT INCLUDE PRENATAL)

◯ None
◯ Less than 1/2 hour per week
◯ 1/2 to almost 1 hour per week
◯ 1 to almost 2 hours per week
◯ 2 to almost 3 hours per week
◯ 3 or more hours per week

Dancing

◯ None
◯ Less than 1/2 hour per week
◯ 1/2 to almost 1 hour per week
◯ 1 to almost 2 hours per week
◯ 2 to almost 3 hours per week
◯ 3 or more hours per week

Yoga, Pilates, stretching, core strengthening exercises, or yoga/pilates/stretching exercise videos or computer games

◯ None
◯ Less than 1/2 hour per week
◯ 1/2 to almost 1 hour per week
◯ 1 to almost 2 hours per week
◯ 2 to almost 3 hours per week
◯ 3 or more hours per week

Cardiovascular exercise machines (treadmill, stationary bike, elliptical, stair climber, rowing machine)

◯ None
◯ Less than 1/2 hour per week
◯ 1/2 to almost 1 hour per week
◯ 1 to almost 2 hours per week
◯ 2 to almost 3 hours per week
◯ 3 or more hours per week

Aerobic exercise classes, aerobic exercise videos or computer games

◯ None
◯ Less than 1/2 hour per week
◯ 1/2 to almost 1 hour per week
◯ 1 to almost 2 hours per week
◯ 2 to almost 3 hours per week
◯ 3 or more hours per week

Weight lifting, resistance exercises

◯ None
◯ Less than 1/2 hour per week
◯ 1/2 to almost 1 hour per week
◯ 1 to almost 2 hours per week
◯ 2 to almost 3 hours per week
◯ 3 or more hours per week

Team sports (volleyball, basketball, softball, etc.)

◯ None
◯ Less than 1/2 hour per week
◯ 1/2 to almost 1 hour per week
◯ 1 to almost 2 hours per week
◯ 2 to almost 3 hours per week
◯ 3 or more hours per week

Please fill out the next section if you work for wages, as a volunteer, or if you are a student.

Are you a homemaker, out of work, or unable to work?

◯ Yes
◯ No

At Work...

Since the beginning of your pregnancy, how much time did you usually spend:

Sitting at work or in class

◯ None
◯ Less than 1/2 hour per day
◯ 1/2 to almost 2 hours per day
◯ 2 to almost 4 hours per day
◯ 4 to almost 6 hours per day
◯ 6 or more hours per day

Standing or slowly walking at work not carrying anything

◯ None
◯ Less than 1/2 hour per day
◯ 1/2 to almost 2 hours per day
◯ 2 to almost 4 hours per day
◯ 4 to almost 6 hours per day
◯ 6 or more hours per day

Standing or slowly walking at work while carrying things (heavier than a 1 gallon milk jug)

◯ None
◯ Less than 1/2 hour per day
◯ 1/2 to almost 2 hours per day
◯ 2 to almost 4 hours per day
◯ 4 to almost 6 hours per day
◯ 6 or more hours per day

Walking quickly at work while carrying things (heavier than a 1 gallon milk jug)

◯ None
◯ Less than 1/2 hour per day
◯ 1/2 to almost 2 hours per day
◯ 2 to almost 4 hours per day
◯ 4 to almost 6 hours per day
◯ 6 or more hours per day

Walking quickly at work not carrying anything

◯ None
◯ Less than 1/2 hour per day
◯ 1/2 to almost 2 hours per day
◯ 2 to almost 4 hours per day
◯ 4 to almost 6 hours per day
◯ 6 or more hours per day

Since the beginning of your pregnancy, when have you usually gotten up in the morning on weekdays?

____

Since the beginning of your pregnancy, when have you usually gotten up in the morning on weekends?

____

Section C: This section asks about symptoms during pregnancy.

Select the answer that best suits your situation since the beginning of your pregnancy.

On average in a day, for how long do you feel nauseated or sick to your stomach?

◯ Not at all
◯ < 1 hr
◯ 2-3 hr
◯ 4-6 hr
◯ >6 hr

On average in a day, how many times do you vomit or thrown up?

◯ I did not throw up
◯ 1-2 times
◯ 3-4 times
◯ 5-6 times
◯ ≥7 times

On average in a day, how many times do you have retching or dry heaves without bringing anything up?

◯ None
◯ 1-2 times
◯ 3-4 times
◯ 5-6 times
◯ ≥7 times

Section D: This section asks about your current behaviors.

Since the beginning of your pregnancy, have you smoked (e.g. tobacco in a traditional cigarette) or used any products (e.g. e-cigarette, vape pen) that contain nicotine? (select all that apply)

☐ Yes, I smoked traditional cigarettes, cigars or used a hookah
☐ Yes, I used e-cigarettes or other vaping devices (e.g. vape pen, e-hookah, etc.)
☐ No

Since the beginning of your pregnancy, how often have you used traditional cigarettes, cigars or a hookah?

◯ Monthly or less
◯ Weekly
◯ Daily

Since the beginning of your pregnancy, how many cigarettes did you smoke on an average day?

____
(cigarettes per day)

Since the beginning of your pregnancy, how often have you used e-cigarettes or other vaping devices?

◯ Monthly or less
◯ Weekly
◯ Daily

Since the beginning of your pregnancy, on days that you used e-cigarettes or other vaping devices, how many times per day did you use them?

____
(times per day)

Since the beginning of your pregnancy, have you used marijuana/cannabis?

◯ Yes
◯ No

Since the beginning of your pregnancy, how often have you used marijuana/cannabis?

◯ Monthly or less
◯ Weekly
◯ Daily

Since the beginning of your pregnancy, on days you used marijuana/cannabis, how many times per day did you use it?

____
(times per day)

Please mark all of the ways you used marijuana/cannabis since the beginning of your pregnancy

- ☐ Smoke (for example, in a joint, bong, pipe, blunt)
- ☐ Vape (for example, in an e-cigarette-like vaporizer or another vaporizing device)
- ☐ Edible/oral (for example, in brownies, cakes, cookies, or candy)
- ☐ Drink (for example, in tea, cola, or alcohol)
- ☐ Dabs/Wax (for example, using waxes or concentrates)
- ☐ Other
- ☐ I do not wish to answer

Please specify:

____

Since the beginning of your pregnancy, during a typical week (Monday through Sunday), how many days per week would you estimate that you consumed an alcoholic beverage? This includes wine, beer, or liquor.

- ◯ Never
- ◯ 1-2 days
- ◯ 3-4 days
- ◯ 5-6 days
- ◯ Every day

On the days when you drank any alcoholic beverages, how many standard drinks did you have on average? One standard drink is equivalent to a 12-ounce beer, a 5-ounce glass of wine, or a drink with one ounce (shot) of liquor.

____
(drinks)

Since the beginning of your pregnancy, what was the greatest number of alcoholic drinks that you consumed in a single day?

____
(drinks)

Section E: This section asks about your social support.

We are interested in how you feel about the following statements. Read each statement carefully. Indicate how you feel about each statement.

	Very Strongly Disagree	Strongly Disagree	Mildly Disagree	Neutral	Mildly Agree	Strongly Agree	Very Strongly Agree
There is a special person who is around when I am in need.	○	○	○	○	○	○	○
There is a special person with whom I can share my joys and sorrows.	○	○	○	○	○	○	○
My family really tries to help me.	○	○	○	○	○	○	○
I get the emotional help and support I need from my family.	○	○	○	○	○	○	○
I have a special person who is a real source of comfort to me.	○	○	○	○	○	○	○
My friends really try to help me.	○	○	○	○	○	○	○
I can count on my friends when things go wrong.	○	○	○	○	○	○	○
I can talk about my problems with my family.	○	○	○	○	○	○	○
I have friends with whom I can share my joys and sorrows.	○	○	○	○	○	○	○
There is a special person in my life who cares about my feelings.	○	○	○	○	○	○	○
My family is willing to help me make decisions.	○	○	○	○	○	○	○
I can talk about my problems with my friends.	○	○	○	○	○	○	○

Section F: The following questions ask you about your feelings and thoughts during the past month.

In the past month, how often have you...

	Never	Almost Never	Sometimes	Fairly Often	Very Often
Been upset because of something that happened unexpectedly?	○	○	○	○	○
Felt that you were unable to control the important things in life?	○	○	○	○	○
Felt nervous and "stressed"?	○	○	○	○	○
Felt confident about your ability to handle your personal problems?	○	○	○	○	○
Felt that things were going your way?	○	○	○	○	○
Found that you could not cope with all the things that you had to do?	○	○	○	○	○
Been able to control irritations in your life?	○	○	○	○	○
Felt that you were on top of things?	○	○	○	○	○
Been angered because of things that were outside of your control?	○	○	○	○	○
Felt difficulties were piling up so high that you could not overcome them?	○	○	○	○	○

Section G: This section asks about your feelings and behaviors over the last 2 weeks. Please answer these questions about yourself as best as you can.

Over the last 2 weeks, how often have you been bothered by any of the following problems?

	Not at all	Several days	More than half the days	Nearly every day
Little interest or pleasure in doing things	○	○	○	○
Feeling down, depressed, or hopeless	○	○	○	○
Trouble falling or staying asleep, or sleeping too much	○	○	○	○
Feeling tired or having little energy	○	○	○	○
Poor appetite or overeating	○	○	○	○
Feeling bad about yourself, or that you are a failure, or have let yourself or your family down	○	○	○	○
Trouble concentrating on things, such as reading the newspaper or watching television	○	○	○	○
Moving or speaking so slowly that other people could have noticed. Or the opposite - being so fidgety or restless that you have been moving around a lot more than usual	○	○	○	○

	Not difficult at all	Somewhat difficult	Very difficult	Extremely difficult
If you checked off any problems, how difficult have these problems made it for you to do your work, take care of things at home, or get along with other people?	○	○	○	○

Section H: This section asks you about ways you may have felt or behaved in the last 2 weeks. Please answer these questions about yourself as best as you can.

Over the last 2 weeks, how often have you been bothered by the following problems?

	Not at all	Several days	More than half the days	Nearly every day
Feeling nervous, anxious, or on edge	○	○	○	○
Not being able to stop or control worrying	○	○	○	○
Worrying too much about different things	○	○	○	○
Trouble relaxing	○	○	○	○
Being so restless that it's hard to sit still	○	○	○	○
Becoming easily annoyed or irritable	○	○	○	○
Feeling afraid as if something awful might happen	○	○	○	○

	Not difficult at all	Somewhat difficult	Very difficult	Extremely difficult
If you checked off any problems, how difficult have these made it for you to do your work, take care of things at home, or get along with other people?	○	○	○	○

Section I: This section asks about your quality of life.

	Not at all	A little	Moderately	Mostly	Completely
To what extent do you feel that your PHYSICAL changes associated with this pregnancy do not allow you to do what you need?	◯	◯	◯	◯	◯
To what extent do you feel that your PSYCHOLOGICAL changes associated with this pregnancy do not allow you to do what you need?	◯	◯	◯	◯	◯
How worried are you about not being able to handle household chores?	◯	◯	◯	◯	◯
How worried are you about carrying out the pregnancy successfully?	◯	◯	◯	◯	◯
How worried are you about not being able to handle labor and delivery?	◯	◯	◯	◯	◯
Have you been forced to cut down on your physical activity during this pregnancy?	◯	◯	◯	◯	◯
How satisfied are you with your partner now?	◯	◯	◯	◯	◯
How satisfied are you with your social life now?	◯	◯	◯	◯	◯
How satisfied are you with how you manage to adapt to this pregnancy?	◯	◯	◯	◯	◯

Section J: This section asks some general questions about yourself.

Gender identity is how someone feels about their own gender. There are many ways a person can describe their gender identity and many labels a person can use. How would you describe your current gender identity? (select all that apply)

- ☐ Woman
- ☐ Man
- ☐ Nonbinary, genderfluid, or genderqueer
- ☐ I am not sure or questioning
- ☐ Some other way

Please specify: ______________________________

How would you describe your race or ethnicity? (check all that apply)

- ☐ Asian Indian
- ☐ Black/African American
- ☐ Cambodian
- ☐ Central American
- ☐ Chinese
- ☐ Cuban
- ☐ Filipino/a/x
- ☐ Inuit/Eskimo/Aleut
- ☐ Japanese
- ☐ Korean
- ☐ Mexican or Mexican American
- ☐ Middle Eastern (Arab, Israeli)
- ☐ Native American/American Indian
- ☐ Native Hawaiian/Pacific Islander
- ☐ Puerto Rican
- ☐ South American
- ☐ Vietnamese
- ☐ White
- ☐ Other

Please specify:

____

What is the highest grade or year of school that you completed?

- ◯ < 12th grade or GED equivalent
- ◯ 12th grade or GED equivalent
- ◯ Trade/technical school
- ◯ 1-3 years of college
- ◯ 4 or 5 years of college
- ◯ 1 year of graduate school
- ◯ 2 or more years of graduate school
- ◯ I don't know
- ◯ I do not wish to answer

What is your current employment status?

- ◯ Employed full time and currently working
- ◯ Employed part time and currently working
- ◯ Student
- ◯ Homemaker/stay at home parent
- ◯ On maternity or family leave from work
- ◯ Unemployed and actively looking for work
- ◯ Unemployed and not looking for work
- ◯ Other

Please specify:

____

What is your total annual household income before taxes?

◯ Less than $50,000 per year
◯ $50,000 to $64,999 per year
◯ $65,000 to $79,999 per year
◯ $80,000 to $99,999 per year
◯ $100,000 to $149,999 per year
◯ $150,000 or greater per year
◯ I don't know
◯ I do not wish to answer

Including yourself, how many people are supported by this income?

____

What is your current marital status?

◯ Married/civil union
◯ Not married, living with a partner
◯ Divorced
◯ Separated
◯ Widowed
◯ Single

What is your wrist size? Please measure the part of your wrist where you would wear a watch as shown in the image below.

__________________________________ (inches)

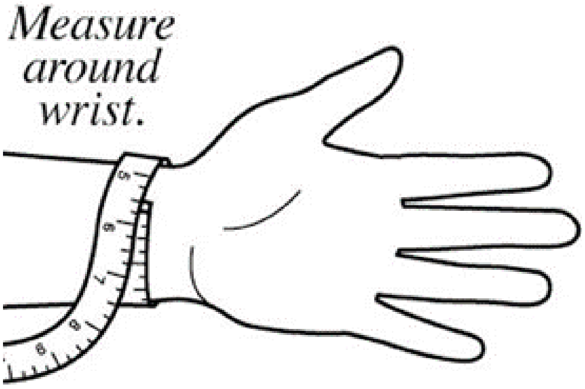

Section K: Contact Information

Please confirm if the contact information we have for you is correct and provide corrections if necessary. We will be using this information to send you the accelerometer and future study surveys.

Email: [participant_info_arm_1][email]
- ◯ Yes
- ◯ No

Please type correct email: ______________________

Phone Number: [participant_info_arm_1][phone]
- ◯ Yes
- ◯ No

Please type correct phone number: ______________________

Address: [participant_info_arm_1][address]
- ◯ Yes
- ◯ No

Please type correct address: ______________________

Do you foresee any problems in picking up the package and wearing the physical activity tracker in a timely manner?
- ◯ Yes
- ◯ No

Is there an alternative address to mail the package? ______________________

Thank you for taking the time to complete this survey. Your participation is greatly appreciated.

If you have any questions, you may contact us at 24MOVEStudy@kp.org or (833) 796-2557.

Feedback Survey

24-Hour Movement and Pregnancy Health (24MOVE) StudyThank you for participating in the 24MOVE Study. This survey includes some questions about your experience in the study. We appreciate you answering the questions honestly so we can improve study procedures for future studies like this. There are no right or wrong answers. If you have any questions, you may contact us at 24MOVE@kp.org or (833) 796-2557.

What was your reason for participating in this research study?

Did the recruitment and informed consent documents prepare you for what to expect as a participant in this research study?

◯ Yes
◯ No

What information would you have liked to know during the recruitment and consent process about what to expect as a participant in this research study?

Were the instructions for wearing the watch easy to understand and follow?

◯ Yes
◯ No

What improvements would you suggest to make the instructions for wearing easier to follow and understand?

Did you have any technical issues with the watch during the study?

◯ Yes
◯ No

Did you find the watch comfortable to wear during the day?

◯ Yes
◯ No

Did you find the watch comfortable to wear at night?

◯ Yes
◯ No

Did you like wearing the watch?

◯ Yes
◯ No
◯ Neutral

What other feedback, if any, would you like to give us about using and wearing the watch?

Were the questions about your previous night's sleep asked by text message (or email) easy to respond to?

◯ Yes
◯ No

What improvements would you suggest to make the questions about your previous night's sleep asked by text message (or email) easier to respond to?

What other feedback, if any, would you like to give us about the sleep surveys that were asked using text messages (or email)?

What other feedback, if any, would you like to give us about your overall study experience?

What questions or uncertainties do you have related to physical activity, sleep, and your pregnancy? What things do you want to know that your health care provider couldn't answer for you? What questions do you think need to be investigated related to physical activity, sleep, and pregnancy to improve healthcare during pregnancy?

Thank you for taking the time to complete this survey. Your participation is greatly appreciated. We will send your Target e-gift card in a separate email.

If you have any questions, you may contact us at 24MOVEStudy@kp.org or 833-796-2557.
